# A feasible strategy for preventing blood clots in critically ill patients with acute kidney injury (FBI): study protocol for a randomized controlled trial

**DOI:** 10.1186/1745-6215-15-226

**Published:** 2014-06-13

**Authors:** Sian Robinson, Aleksander Zincuk, Ulla Lei Larsen, Claus Ekstrøm, Palle Toft

**Affiliations:** 1 Department of Anesthesia and Intensive Care, Odense University Hospital, Sdr. Boulevard 29, Odense C DK 5000, Denmark; 2 Department of Biostatistics, University of Copenhagen, Øster Farimagsgade 5, Copenhagen K DK-1014, Denmark

**Keywords:** Enoxaparin, Thromboprophylaxis, Critically ill patients, Anti-factor Xa activity, Deep vein thrombosis, Pulmonary embolism, Acute kidney injury, Continuous renal replacement therapy, Neutrophil gelatinase-associated lipocalin, Renal recovery

## Abstract

**Background:**

Previous pharmacokinetic trials suggested that 40 mg subcutaneous enoxaparin once daily provided inadequate thromboprophylaxis for intensive care unit patients. Critically ill patients with acute kidney injury are at increased risk of venous thromboembolism and yet are often excluded from these trials. We hypothesized that for critically ill patients with acute kidney injury receiving continuous renal replacement therapy, a dose of 1 mg/kg enoxaparin subcutaneously once daily would improve thromboprophylaxis without increasing the risk of bleeding. In addition, we seek to utilize urine output prior to discontinuing dialysis, and low neutrophil gelatinase-associated lipocalin in dialysis-free intervals, as markers of renal recovery.

**Methods/Design:**

In a multicenter, double-blind randomized controlled trial in progress at three intensive care units across Denmark, we randomly assign eligible critically ill adults with acute kidney injury into a treatment (1 mg/kg enoxaparin subcutaneously once daily) or control arm (40 mg enoxaparin subcutaneously once daily) upon commencement of continuous renal replacement therapy.

We calculated that with 133 patients in each group, the study would have 80% power to show a 40% reduction in the relative risk of venous thromboembolism with 1 mg/kg enoxaparin, at a two-sided alpha level of 0.05. An interim analysis will be conducted after the first 67 patients have been included in each group.

Enrolment began in March 2013, and will continue for two years. The primary outcome is the occurrence of venous thromboembolism. Secondary outcomes include anti-factor Xa activity, bleeding, heparin-induced thrombocytopenia, filter lifespan, length of stay, ventilator free days, and mortality. We will also monitor neutrophil gelatinase-associated lipocalin and urine volume to determine whether they can be used as prognostic factors for renal recovery.

**Discussion:**

Critically ill unit patients with acute kidney injury present a particular challenge in the provision of thromboprophylaxis. This study hopes to add to the growing evidence that the existing recommendation of 40 mg enoxaparin is inadequate and that 1 mg/kg is both safe and effective for thromboprophylaxis.

In addition, the study seeks to identify predictors of renal recovery allowing for the proper utilization of resources.

**Trial Registration:**

EU Clinical Trials Register: EudraCT number: 2012-004368-23, 25 September 2012.

## Background

Approximately 30% of critically ill patients admitted to an intensive care unit (ICU) develop acute kidney injury (AKI) [1]. Biomarker neutrophil gelatinase-associated lipocalin (NGAL) is associated with the development of AKI, the need for dialysis, and mortality [1-4].

Despite ICU patients receiving recommended doses of prophylactic low- molecular-weight heparin (LMWH), between 5 and 15.5% develop proximal leg deep-vein thrombosis (DVT) [5,6]. ICU patients with AKI frequently have activated coagulation pathways triggered by increased tissue factor and fibrinogen, reduced fibrinolysis, activation of endothelial surfaces, and thrombocytes. Thus such patients have a four-fold higher risk for developing venous thromboembolism (VTE) compared with ICU patients without renal insufficiency [7-9]. Critically ill patients with AKI are also predisposed to bleeding because of uremic platelet dysfunction, coagulopathies, and comorbidities. However many bleeding episodes are deemed as minor [10,11].

Major or fatal bleeding is very rare in the ICU, whereas VTE is relatively more common. VTE may not be detected in the ICU patient population as the symptoms are often minimal or atypical [12-14]. In many studies high-dose enoxaparin yields high peak and trough anti-factor Xa (anti-X_a_) values, but no increase in bleeding complications regardless of the degree of renal insufficiency [15]. A delay in starting thromboprophylaxis was associated with an increased risk of mortality in patients on the ICU [16]. Thus, the risk-benefit ratio seems to favor use of anticoagulant thromboprophylaxis [10].

In a recent study, we showed that a weight-based dose of enoxaparin subcutaneous (sc) once daily (QD) contrary to the standard dose of 40 mg enoxaparin sc QD yielded satisfactory levels of anti-X_a_ for critically ill patients, was more likely to maintain anti-X_a_ levels within the therapeutic range for longer periods of time and did not result in bioaccumulation [17].

The primary objective of this study is to reduce the incidence of VTE among AKI patients on continuous renal replacement therapy (CRRT) by using 1 mg/kg enoxaparin sc QD, versus the standard dose of 40 mg enoxaparin sc QD. We hypothesized that higher doses of enoxaparin would optimize thromboprophylaxis without increasing the risk of bleeding in these patients. Secondary objectives of the trial include examining the utility of neutrophil gelatinase-associated lipocalin (NGAL) levels and urine output as predictors of renal recovery.

## Methods/Design

### Trial design

This is a multicenter randomized controlled trial conducted at three ICUs across Denmark to assess whether a dose of 1 mg/kg enoxaparin versus standard dose of 40 mg enoxaparin will improve thromboprophylaxis in AKI patients on CRRT, without increasing the risk of bleeding. We will randomly assign eligible critically ill adults with AKI into a treatment arm (1 mg/kg enoxaparin sc QD) or a control arm (40 mg enoxaparin sc QD) upon commencement of CRRT. The sample population will consist of 133 patients from ICUs at Odense University Hospital (OUH), Svendborg Hospital, and SVS Esbjerg Hospital. An interim analysis will be conducted after the first 67 patients have been included in each group. Enrolment began in March 2013 and will continue until March 2015. Patients will be followed until either: (1) a combination of VTE and renal recovery, (2) a combination of heparin - induced thrombocytopenia (HIT) and renal recovery, (3) ICU death or discharge, or (4) change from continuous to intermittent dialysis. Investigators will obtain consent for the collection of data on treatments and outcomes for participants who withdraw prematurely from the project. Many of the current investigators have participated in other trials and, as such, have a proven track record with respect to enrolment and follow-up of participants and collecting complete data sets. We will document the reasons for missing data, collect auxiliary variables that may predict outcome and/or determine which patients are more susceptible to dropout, and follow-up on all such patients until 24 hours after the last dose of enoxaparin.

### Ethics

Ethical approval for this protocol was obtained from the Danish national scientific ethical committee (reference number: 1210528) and the study was also approved by Danish Health and Medicines Authority (reference number: 2012100176) (see Tables 1 and 2). The study will comply with the ethical principles set forth in the Declaration of Helsinki and will be conducted in accordance with good clinical practice. Funding sources for this research had no role in the design of this study and will have no authority over data collection, analysis, interpretation, or reporting of this study.

**Table 1:** World Health Organization trial registration data set

**Data category**	**Information**
Primary registry and trial identifying number	EU clinical trials register:
	EudraCT number: 2012-004368-23
Date of registration in primary registry	25/09/2012
Secondary identifying numbers	Danish health and medicines authority: 2012100176
	Danish national scientific ethical committee: 1210528 Sponsor's protocol number 20121005
Source(s) of monetary or material support	Danish society of anesthesiology & intensive medicine’s research initiative
	Odense University Hospital’s research grant
	Lippmann fund
Primary sponsor	Palle Toft, Professor at the department of Anesthesiology and Intensive Care at Odense University Hospital, Odense, Denmark.
Secondary sponsor(s)	N/A
Contact for public queries	Sian Robinson, MB, BS; EDIC
	Odense University Hospital
	Department of Anesthesiology and Intensive Care
	Sdr. Boulevard 29. Odense C
	DK 5000. Denmark
	Telephone: +45 6541 5519
	Email: sian.robinson@rsyd.dk
Contact for scientific queries	Sian Robinson, MB, BS; EDIC
	Principal investigator
	Odense University Hospital
	Department of Anesthesiology and Intensive Care
	Sdr. Boulevard 29. Odense C
	DK 5000. Denmark
	Telephone: +45 6541 5519
	Email: sian.robinson@rsyd.dk
Public title	A feasible strategy for preventing blood clots in critically ill patients with acute kidney injury (FBI)
Scientific title	A feasible strategy for preventing blood clots in critically ill patients with acute kidney injury (FBI) - prospective randomized, double-blind multicenter study
Countries of recruitment	Denmark
Health condition(s) or problem(s) studied	Venous thromboembolism, enoxaparin dose, acute kidney injury, bleeding
Intervention(s)	Treatment arm: 1 mg/kg enoxaparin subcutaneous once daily
	Control arm: 40 mg enoxaparin subcutaneous once daily
	Enoxaparin will be administered subcutaneously to the thigh or abdomen of the study patients from the day of inclusion, until the end of each participant’s study period.
Key inclusion and exclusion criteria	Inclusion criteria: patients are eligible if they give consent, develop acute kidney injury, need continuous renal replacement therapy, weigh 45 to 150 kg, and are ≥18 years.
	Exclusion criteria: these include a) admission diagnosis of major trauma, b) need for therapeutic anticoagulation, c) contraindication to heparin (allergy or heparin-induced thrombocytopenia), d) pregnancy, e) life-support limitation, f) uncontrolled hypertension (bp > 180/110) for at least 12 hours, g) cerebral hemorrhage/acute gastrointestinal bleed, h) severe thrombocytopenia (platelet count <50 × 109/l), i) International Normalized Ratio (INR) or activated partial thromboplastin time (APTT) ≥2 times the upper limit of normal, j) chronic renal failure or acute-on-chronic renal failure, and k) initial evaluation more than 24 hours after commencement of continuous renal replacement therapy.
Study type	Interventional allocation: randomized
	Intervention model: double arm
	Masking: double blind (subject, relatives, investigator, outcomes assessor)
	Primary purpose: prevention
Date of first enrolment	March 2013
Target sample size	266
Recruitment status	Recruiting
Primary outcome(s)	Venous thromboembolism
Key secondary outcomes	Catheter-related thrombus, anti-Xa activity, bleeding, heparin-induced thrombocytopenia, filter lifespan, length of stay, ventilator free days, and mortality. We will monitor neutrophil gelatinase-associated lipocalin levels and urine volume to determine whether they can be used as prognostic factors for renal recovery.

**Table 2:** Protocol revision chronology

**Date**	**Protocol amendment**
Original version, 22.02.2011	Amendment Number 1:
	Primary reason for amendment:
	Changes in the methods sections - a decision to allow the pharmacy at OUH to generate randomization code was made.
Version 2, 18.05.2011	Amendment Number 2:
	Primary reason for amendment:
	At the request of GCP changes in the ethics section to elaborate on the procedure for obtaining consent.
Version 3, 18.07.2012	Amendment Number 3:
	Primary reason for amendment:
	The methods section was amended to include a description of the conduct of CRRT during the trial.
Version 4, 25.08.2012	Amendment Number 4:
	Primary reason for amendment:
	Fleming-Harrington-O’Brien stopping rule and futility measure via ’conditional power’ calculation was introduced.
Version 5, 31.08.2012	Amendment Number 5:
**Version 5 was the first version to be submitted to the Danish Health and Medicines Authority and the Danish national scientific ethical committee.**	Primary reason for amendment:
	The comparator dose which was unconfirmed until this point was included in this new protocol version.
	At the request of Danish Health and Medicines Authority changes were made to the exclusion criteria so that patients with chronic renal failure or acute-on-chronic renal failure were ineligible. The methods section was also updated to indicate that a patient who changed from CRRT to intermittent hemodialysis would have reached the end of the study period. In addition, further details about the reporting of adverse events were included.
Version 6, 21.01.2013	Amendment Number 6:
	Primary reason for amendment:
	At the request of the Danish national scientific ethical committee a separate information sheet for the designated surrogates was developed.
	The methods section was also revised due to the acquisition of new dialysis machines at OUH.
Version 7, 06.06.2013	Amendment Number 7:
	Primary reason for amendment:
	A change in the exclusion criteria:
	platelet count of <75× 109/l, changed to <50 × 109/l and INR or APTT ≥1½ times the upper limit of normal changed to ≥2 times the upper limit of normal.

Issue date: 06 June 2013. Protocol Amendment Number: 07. Authors: Sian Robinson, MB BS; EDIC; Aleksander Zincuk, MD; Ulla Lei Larsen, MD; Claus Ekstrøm, PhD; Palle Toft, MD, DMSc. OUH, Odense University Hospital; GCP, Good Clinical Practice; CRRT, Continuous renal replacement therapy; APTT, Activated partial thromboplastin time; INR, International normalized ratio.

Research physicians on the ICUs of the aforementioned three hospitals will obtain written informed consent from all potential trial participants or their designated surrogates for participation in the study. The participants will then be randomized following recruitment and clinical interviews. For those who suffer harm from trial participation, there is the possibility to appeal and receive compensation according to the Act on the Right to Complain and Receive Compensation within the Danish health service. At the end of the trial, participants who indicate on the consent form that they wish to be informed of the results will be contacted by the trial sponsor. The model consent form and other related documents given to participants and designated surrogates are provided as supplements to this protocol (Additional file 1, Additional file 2, and Additional file 3).

All laboratory specimens, reports, data forms, and other study-related information are identified by a coded number only. These are stored separately from participant-related information, both in locked file cabinets in areas with limited access. The web-based FBI database (Department of Health Cooperation and Quality Database Unit, Region of Southern Denmark, Vejle, Denmark) which houses the study’s data collection forms is password-protected. The project principal investigators only have direct access to their own site’s data sets.

Records of each participant's identity and personal information will not be made public. Employees from Good Clinical Practice (GCP), Danish Health and Medicines Authority, and the corresponding foreign authority will have access to participants’ medical records and information collected in connection with the study for quality control (monitoring, audit, and inspection) of the project. GCP is independent from the sponsor and competing interests and will serve as a liaison between all three hospitals and the data manager, ensuring continuity. GCP has direct access to all three sites’ data sets and as such, will work with the data manager to oversee the intra-study data sharing process. A Data Monitoring Committee (DMC) with Bjarne Dahler-Eriksen MD, PhD as chair (ICU), Torben Bjerregaard Larsen MD, PhD (centre for thrombosis), Helle Asboe Jørgensen MD, EDIC (ICU), and Jacob von Bornemann Hjelmborg MSc, PhD (statistician) has been established. The DMC is independent of those conducting, sponsoring, or funding the FBI trial. The DMC members are required to indicate their assent, as well as to declare any competing interests in the FBI charter. This charter which details the DMC’s roles and responsibilities, and planned method of functioning is available on request from the FBI study office.

### Study participants

Study participants will comprise two groups of critically ill patients with AKI on CRRT.

### Inclusion criteria

Patients are eligible for inclusion if they give consent, develop AKI, need CRRT, weigh between 45 and 150 kg, and are more than or equal to 18-years-old.

### Exclusion criteria

These include: a) admission diagnosis of major trauma, b) need for therapeutic anticoagulation, c) contraindication to heparin-allergy or HIT, d) pregnancy, e) life-support limitation, f) uncontrolled hypertension (blood pressure >180/110) for at least 12 hours, g) cerebral hemorrhage or acute gastrointestinal bleeding, h) severe thrombocytopenia (platelet count <50 × 109/l), i) International Normalized Ratio (INR) or activated partial thromboplastin time (APTT) more than or equal to two times the upper limit of normal, j) chronic renal failure or acute-on-chronic renal failure, and k) initial evaluation after more than 24 hours have elapsed from commencement of CRRT.

### Randomization

**Figure 1:**
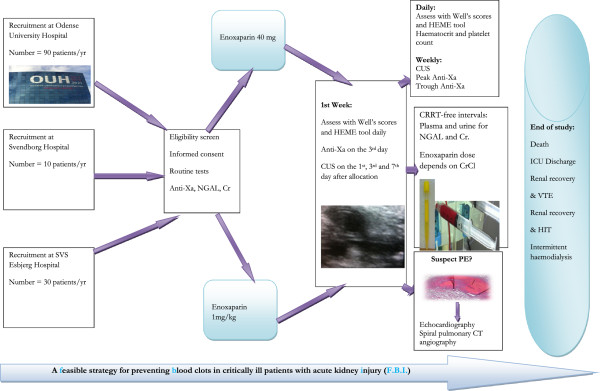
**Time schedule of enrolment, interventions, and assessments for participants.** Routine tests (APTT, platelets, D-dimer, AT, leukocytes, CRP, PCT, hematocrit), anti-Xa, and urine and plasma NGAL and Cr are measured at baseline. Following randomization, the patient will receive sc enoxaparin according to group allocation. Bedside clinical assessment for VTE and bleeding using validated scoring systems (Well's scores, HEME tool) will be conducted, and samples for hematocrit and platelet count will be taken daily. Bilateral lower extremity CUS will be conducted on the 1st, 3rd and 7th day of inclusion. Once weekly CUS (more frequent if DVT is suspected), as well as samples for peak anti-X_a_ (measured at 4 hours after enoxaparin dose) and trough anti-X_a_ (measured at 20 hours after enoxaparin dose) will be taken. Spiral pulmonary CT angiography and echocardiography will be performed on clinical suspicion of PE. During CRRT-free intervals with CrCl <30 ml/min/1.73 m^2^, group allocation will be discontinued and participants will receive 40 mg enoxaparin QD until the CrCl is >30 ml/min/1.73 m^2^ when enoxaparin dose according to group allocation will be resumed. Samples of urine and plasma will be taken for NGAL and Cr during CRRT-free intervals. APTT, Activated partial thromboplastin time; anti-Xa, Antifactor Xa; AT, Antithrombin; CRP, C-reactive protein; PCT, Procalcitonin; NGAL, Neutrophil gelatinase-associated lipocalin; Cr, Creatinine; sc, subcutaneous; VTE, Venous thromboembolism; CUS, Compression ultrasound; DVT, Deep venous thrombosis; CT, Computed tomography; PE, Pulmonary emboli; CRRT, Continuous renal replacement therapy; CrCl, Creatinine Clearance; QD, Once daily; HIT, Heparin-induced thrombocytopenia; ICU, Intensive care unit.

All patients who fulfil the inclusion criteria and who give consent for participation will be randomized. The randomization sequence was generated in block sizes of four by the hospital’s pharmacy at OUH, using a web-based randomization system. This allocation sequence is being implemented through use of sequentially numbered, opaque, sealed envelopes. Each center will screen subjects to achieve the enrolment targets demonstrated in Figure 1. In the event of failure to meet target population, the study will be expanded to other sites. Allocation concealment will be ensured as the randomization code will not be revealed until the patient has been recruited into the trial.

### Blinding

The medicine module of Critical Information System (CIS LIVE™; Daintel, Copenhagen, Denmark) used at all three hospitals allows for blinding. Patients, family members, clinicians, research personnel, radiologists, laboratory technicians, and the trial biostatistician will all be unaware of study-group assignments. They will remain blinded until the study database is locked at the end of the trial. Nurses who administer the drug will be the only party privy to the actual dose given to each patient as it is impossible to prepare an enoxaparin dose of 1 mg/kg beforehand. In the event of an emergency (major bleeding, need for major surgery), a participant’s allocated intervention may be revealed to the attending duty physician by the nurse providing care for that participant. The actual allocation will not be disclosed to the patient or study personnel, and there will be no disclosure of the code in any of the corresponding patient documents.

### Interventions

The intervention for the treatment arm will be 1 mg/kg enoxaparin sc QD, and the intervention for the control arm will be 40 mg enoxaparin sc QD. Enoxaparin will be administered to the thigh or abdomen of the study patients.

### Dispensing

Enoxaparin (Klexane™; Sanofi-Aventis Denmark A/S, Hørsholm, Denmark) 100 mg/ml is available as prefilled single-dose syringes containing 40 mg, or as multi-dose vials containing 300 mg in 3 ml. Nurses will carefully titrate a dose of 1 mg/kg, using 1 ml or 2 ml syringes as needed.

### Modifications

Project medicine will be adjusted in several situations. If creatinine-clearance (CrCl) is <30 ml/min/1.73 m^2^ during CRRT-free intervals, group allocation will be discontinued and participants will receive 40 mg QD. Once CrCl is >30 ml/min/1.73 m^2^, group allocation will be resumed. In the event of VTE, group allocation will be discontinued, and patients will receive a full treatment dose of enoxaparin. Enoxaparin will be temporarily discontinued in the case of oozing, and packed red blood cells, fresh frozen plasma, and protamine sulphate may be administered at the discretion of the attending physician. Enoxaparin will be resumed according to the group allocation when oozing is no longer observed. Should the platelet count decrease to less than 50 × 109/L, or if there is an unexplained platelet count decrease to less than 50% of baseline, or other suspicion of HIT, enoxaparin will be stopped and mechanical prophylaxis or the HIT-safe anticoagulant, argatroban(Novastan™; Swedish Orphan Biovitrum A/S, Lyngby, Denmark), will be started. The patient will be evaluated by the 4 T’s clinical scoring system [18] and blood samples will be taken for PF4/heparin enzyme-linked immunosorbent assay (ELISA) and 14C-platelet serotonin release assay.

### Adherence

To ensure protocol adherence and data quality, research coordinators attended study-specific training sessions. Throughout the trial participating centers will also educate rotating ICU residents and hold periodic updates for nurses. GCP monitoring will also serve to improve protocol adherence.

All the research physicians who will perform interventions on participants are trained in anaesthesia and intensive care, and as such have great experience with using ultrasound. In addition, all investigators received specific training in the performance of venous compression ultrasound (CUS) of the under extremities. The majority of these physicians also have prior training in Focused Assessed Transthoracic Echocardiography (FATE). All other aspects of care and management of trial outcomes are at the ICU team’s discretion.

### Adverse events

All serious adverse events will be immediately reported to the sponsor. The immediate reports will be followed within 24 hours by detailed written reports (please see Additional file 4). The investigator will also comply with the regulatory requirements related to the reporting of unexpected serious adverse drug reactions to the Danish national scientific ethical committee and the Danish Health and Medicines Authority.

The sponsor will expedite the reporting of all adverse drug reactions that are both serious and unexpected to all concerned investigators and/or institutions, the Danish national scientific ethical committee and the Danish Health and Medicines Authority within the timeframe specified by these authorities.

The sponsor will submit to the regulatory authorities all safety updates as required by these regulatory bodies. The following will not be reported: symptoms related to the patient’s underlying disease (such as fatigue, diarrhoea, pain, nausea, or vomiting), delirium, electrolyte imbalance, and death that ensues because of withdrawal of active treatment.

### Outcome measures

The primary outcome is the occurrence of proximal leg DVT detected 3 days or more after randomization or pulmonary embolism (PE) diagnosed on computed tomography scan (CT) of the chest or at autopsy (for definitions of outcome measures please see Additional file 5). Secondary outcomes include all other DVTs, catheter-related thrombus, anti-Xa activity, bleeding, HIT, filter lifespan, length of stay, ventilator free days, and mortality. In addition, we will monitor NGAL levels and urine volume to determine whether they can be used as prognostic factors for renal recovery.

### Study conduct

This trial is active and enrolment began in March 2013, and will continue until March 2015. The study scheme is outlined in Figure 1. For details on the conduct of CRRT please see Additional file 6. We will record admission diagnosis, ICU severity of illness scores, and baseline demographic data for both groups of participants.

### Examination and blood tests

Anti-Xa, activated partial thromboplastin time (APTT), platelets, D-dimer, antithrombin (AT), leukocytes, c-reactive protein (CRP), procalcitonin (PCT), hematocrit, urine and plasma NGAL, urine and plasma creatinine (Cr) are all measured at baseline. Peak and trough anti-X_a_ will be measured on day three, and once weekly. We will record the urine output prior to discontinuation of dialysis, and measure NGAL and Cr during CRRT-free intervals. All patients will have daily hematocrit and platelet count measured during the study period. Other parameters will be measured as per the departments’ norm. Samples for anti-Xa activity will be analyzed in a blinded fashion using a validated chromogenic assay kit (COAMATIC Heparin, Chromogenix, Instrumentation Laboratory Company, Lexington, Kentucky, United States). NGAL in urine and plasma will be quantified by particle-enhanced turbidimetric immunoassay (NGAL Test™, Roche Modular P apparatus, BioPorto Diagnostics A/S, Gentofte, Denmark) according to the manufacturer’s instructions.

Patients will undergo daily bedside clinical assessment for bleeding or VTE using validated tools for the ICU population [11,19,20]. Bilateral lower extremity CUS at 1-cm intervals from the iliac/common femoral vein to the popliteal vein will be conducted on the first, third, and seventh day of inclusion. CUS will then be repeated on a weekly basis (more frequently if DVT is clinically suspected). All positive CUS will be verified by a radiologist. Serial ultrasound examinations will be terminated if a positive study for DVT is obtained. Spiral pulmonary CT angiography and echocardiography will be performed on clinical suspicion of PE.

Patients will be followed until one of the following occurs: a combination of VTE and renal recovery, a combination of HIT and renal recovery, ICU death or discharge, or a change from continuous to intermittent dialysis.

### Sample size and statistical methods

We hypothesized that the optimal dose of enoxaparin would reduce the incidence of VTE in critically ill AKI patients requiring CRRT without causing an increase in bleeding. An additional hypothesis was that high urine output prior to the discontinuation of dialysis and low NGAL in dialysis-free intervals can predict renal recovery in these patients.

We estimated that with 133 patients in each group the study would have 80% power to show a 40% reduction in the relative risk of VTE with 1 mg/kg enoxaparin sc QD, assuming an incidence rate of 40% in the control group, at a two-sided alpha level of 0.05. An interim analysis (for both safety and efficacy) after approximately 50% of the patients have completed the study will be performed by an independent statistician blinded to the treatment allocation. These outcomes will be analyzed with stopping rules based on the Fleming-Harrington-O’Brien stopping boundary using *P* = 0.01, with adjustment for an overall type 1 error of 0.05, and with the final analysis conducted at *P* = 0.045 [21]. The statistician will report, in strict confidence, to the DMC (see Table 3). The DMC members will not be blinded to patient allocation. They may request other analyses (such as data from other comparable trials) prior to reaching a decision on the continuation of the trial. The DMC will report its recommendations in writing to the Trial Steering Committee. The study can also be terminated if the difference between groups is negligible and the statistician using ’conditional power’ calculation determines that continuation will be futile.

**Table 3:** Organizational structure and responsibilities

**Title**	**Name(s)**	**Function(s)**
Principal investigator and Research Physician	Sian Robinson, MB, BS; EDIC	Design and conduct of trial F.B.I., protocol preparation and revisions, preparation of Case Report Forms, managing Clinical Trials Office, publication of study reports,member of both committees, and organization of steering committee meetings.
Lead investigators	Sian Robinson, MB, BS; EDIC Odense University Hospital	Maintain trial master file and resolve contractual issues at sites
	Stine Zwisler, MD, Phd	
	Svendborg Hospital	
	Karen Doris Boedker, MD SVS Esbjerg Hospital	Responsible for identification, recruitment, data collection, and completion of CRFs, along with follow-up of study patients and adherence to study protocol.
Steering committee	Sian Robinson, MB ,BS; EDIC	Agreement of final protocol
	Aleksander Zincuk, MD	Reviewing progress of study
	Ulla Lei Larsen, MD	Approving changes to the protocol
	Claus Ekstrøm, PhD	Budget administration
	Palle Toft, MD, DMSc	Advice for lead investigators
Trial management committee	Sian Robinson, MB ,BS; EDIC	Decide when site visit will occur
	Stine Zwisler, MD, PhD	Data verification
	Karen Doris Boedker, MD	Randomization
	Aleksander Zincuk, MD	Provide annual risk report to Danish Health and Medicines
	Ulla Lei Larsen, MD	Authority and Danish national scientific ethical committee
	Claus Ekstrøm, PhD	Serious unexpected suspected adverse events (SUSAR) reporting
	Palle Toft, MD, DMSc	
Data manager	Azmir Salihovic	Maintenance of trial IT system and data verification
Data monitoring committee	Bjarne Dahler-Eriksen MD, PhD	Review the interim analysis.
	Torben Bjerregaard Larsen, MD, PhD	Communicate the outcome of its deliberations to the Trial Steering Committee.
	Helle Asboe Jørgensen MD, EDIC	Independent from the sponsor and the other committees.
	Jacob von Bornemann Hjelmborg MSc, PhD	

We will analyze data from all randomized patients according to their assigned group (intention-to-treat principle). We will use either estimating-equation methods or continuous repeated measures (a statistical model) in the event of missing data. All baseline demographic values for these two groups will be compared, using the Student’s *t*-test or Mann-Whitney rank sum test for continuous variables and chi-squared (χ^2^) or Fisher's exact test for categorical variables.

The primary outcome VTE will be compared between the two groups. Four pre-specified subgroup analyses based on: 1) the patient's ICU admission (surgical versus medical), 2) center experience, 3) presence or absence of edema, and 4) presence or absence of vasopressor or inotropes use will be performed.

Multiple regression analysis will conducted to evaluate: 1) the influence of vasopressor, respiratory support, comorbidity, organ dysfunction scores, and CrCl on the incidence of VTE and bleeding between groups; and 2) to examine the relationship between biomarker, demographic variables, comorbidity, and Acute Kidney Injury Network classification.

The prediction ability of urine output and NGAL for successful discontinuation of CRRT will be assessed with the area under the receiver operating characteristic (ROC) curve method. Anti-Xa levels will be analyzed by repeated analysis modelling. For survival analysis, we will calculate hazard ratios and associated 95% confidence intervals using Cox regression analysis and the Kaplan-Meier method with log-rank testing.

## Discussion

LMWHs at standard doses appear to protect ward patients against VTE, but what is less clear is the optimal dose suitable for ICU patients. ICU patients with AKI present a particular challenge because of their increased risk of bleeding and thrombosis. Existing guidelines in Denmark recommend 40 mg enoxaparin for thromboprophylaxis, but this is thought to be inadequate. A previous pharmacokinetic trial suggested that a dose of 1 mg/kg enoxaparin may offer optimal thromboprophylaxis [17]. Our current study aims to confirm 1 mg/kg enoxaparin is the optimal dose in this high-risk population. As the study design is one of a randomized double-blinded multicenter trial, it should be considered a high quality trial aiming to provide class I evidence of the primary endpoint.

So far, only one type of LMWH (dalteparin) has won approval for use in patients with severe renal insufficiency [22]. Critically ill patients have decreased levels of protein, and thus a larger free fraction of enoxaparin. The volume of distribution of enoxaparin is only 5 L and this, coupled with a small molecular weight, allow for normal dosing as the drug would be expected to be cleared by the high volumes of fluid employed in hemofiltration. Thus, our study has implications for clinical practice as our findings may challenge the premise that selected LMWHs should be avoided in such patients.

Finally, there are currently no established guidelines for the discontinuation of dialysis. We seek to use a combination of a clinical indicator (urine volume) and a biomarker (NGAL) to predict renal recovery.

## Trial status

This trial is active. Enrolment began in March 2013 and will continue for two years.

## Abbreviations

APTT: Activated partial thromboplastin time; AKI: Acute kidney injury; anti-Xa: Antifactor Xa; AT: Antithrombin; CRP: C-reactive protein; χ^2^: Chi-squared; CUS: Compression ultrasound; CT: Computed tomography; CRRT: Continuous renal replacement therapy; CVVHD: Continuous veno-venous hemodialysis; CVVH: Continuous veno-venous hemofiltration; Cr: Creatinine; CrCl: Creatinine Clearance; DMC: Data Monitoring Committee; DVT: Deep venous thrombosis; ELISA: Enzyme-linked immunosorbent assay; FATE: Focused Assessed Transthoracic Echocardiography; FBI: Feasible strategy for preventing blood clots in critically ill patients with acute kidney injury; GCP: Good Clinical Practice; HIT: Heparin-induced thrombocytopenia; ICU: Intensive care unit; INR: International normalized ratio; LMWH: Low- molecular-weight heparin; NGAL: Neutrophil gelatinase-associated lipocalin; OUH: Odense University Hospital; QD: Once daily; PCT: Procalcitonin; PE: Pulmonary emboli; ROC: Receiver-operator characteristic; sc: Subcutaneous; UFH: Unfractionated heparin; VTE: Venous thromboembolism.

## Competing interests

The authors declare that they have no competing interests.

## Authors’ contributions

PT conceived the research and SR, with input from PT, CE, AZ, and ULL designed the study, and ensured all important issues of an interventional trial were addressed (please see Additional file 7). SR secured founding for the project, wrote the original and final draft of the manuscript, will recruit and follow patients, interpret the results, and perform the statistical analysis. AZ and ULL will assist with patient enrolment, examination and data collection, in conjunction with appointed principal investigators at the other sites. CE will assist with the statistical analysis. The interval between the completion of data collection and the release of the study results will be kept to an absolute minimum. The study results will be released to the participating physicians, patients and the general medical community. We expect to take about 2 months to compile the final paper for an appropriate English language journal. It will list SR as the first author (who is a native speaker of English) and no professional writers will be used. The Vancouver Protocol for determining authorship will be applied (see Table 3). The results will be disseminated regardless of the statistical significance. There are no restrictions imposed on the investigators’ right to publish or present trial results. All authors read and approved the final manuscript.

## Supplementary Material

Additional file 1 The rights of a trial subject in a biomedical research project. Click here for file

Additional file 2 Information for participants in the scientific trial: a feasible strategy for preventing blood clots in critically ill patients with acute kidney injury (FBI)0. Click here for file

Additional file 3 Informed consent for participation in the biomedical research project: a feasible strategy for preventing blood clots in critically ill patients with acute kidney injury (FBI). Click here for file

Additional file 4 Report form for Serious Adverse Event and Suspected Unexpected Serious Adverse Event. Click here for file

Additional file 5 Definitions. Click here for file

Additional file 6 Conduct of continuous renal replacement therapy. Click here for file

Additional file 7 SPIRIT checklist. Click here for file
