# Supplementary material for: A feasible strategy for preventing blood clots in critically ill patients with acute kidney injury (FBI): study protocol for a randomized controlled trial
Source: Trials. 2014 Jun 13;15:226. doi: 10.1186/1745-6215-15-226 (PMC4061539; doi:10.1186/1745-6215-15-226)
Supplement: Additional file 1 — The rights of a trial subject in a biomedical research project. [file 1745-6215-15-226-S1.pdf]

The rights of a trial subject in a biomedical research project

As a participant in a biomedical research project you should know that:

- your participation in the research project is completely voluntary and can only take place after you have received both written and oral information about the research project and signed the consent form;

- you may at any time orally, in writing or by any other clear notification withdraw your consent to participation and withdraw from the research project. If you withdraw your consent, this will not affect your right to any current or future treatment or any other rights you may have;

- you are entitled to bring a member of your family, a friend or an acquaintance with you to the informative interview;

- you are entitled to time to think it through before you sign the consent form;

- strict confidentiality is observed with regards to information about your health, other purely private matters and other confidential information about you disclosed in connection with the research project;

- information about you, including information about tissue and blood samples from you, will be stored according to the provisions specified in the Danish Act on Processing of Personal Data and the Health Act.

- you will be able to get access to research protocols according to the provisions of the Danish Open Administration Act. This means that you can gain access to all documents concerning your participation in the project apart from the parts containing business secrets or confidential information about others.

- you have the right to complain and compensation can be paid according to the Act on the Right to Complain and Receive Compensation within the Health Service.
