# Supplementary material for: A feasible strategy for preventing blood clots in critically ill patients with acute kidney injury (FBI): study protocol for a randomized controlled trial
Source: Trials. 2014 Jun 13;15:226. doi: 10.1186/1745-6215-15-226 (PMC4061539; doi:10.1186/1745-6215-15-226)
Supplement: Additional file 2 — Information for participants in the scientific trial: a feasible strategy for preventing blood clots in critically ill patients with acute kidney injury (FBI)0. [file 1745-6215-15-226-S2.pdf]

OUH
Odense
Universitetshospital

Region Syddanmark

We would like to request your participation in the scientific trial: A feasible strategy for preventing blood clots in critically ill patients with acute kidney injury (F.B.I.).

This trial is being conducted by:

Sponsor-investigator: Palle Toft, MD, DMSc

Principal investigator: Sian Robinson, M.B., B.S.

Co-investigators: Aleksander Zincuk, MD
Ulla Lei Larsen, MD

Before you decide whether to participate in the trial, you need to be fully informed about the trial and the reasons for conducting the study. We would therefore ask you to read this information for participants thoroughly.

You will be invited to an interview about the trial where we will further elaborate on concepts found in the participant information sheet, and where you will be able to have your queries addressed. You are welcome to bring a family member, friend or acquaintance to the interview, and you are allowed time to reflect on whether you wish to participate or not. You may ask questions at any time in the process.

If you decide to participate in the trial, we will ask you to sign a consent declaration. Remember that you are entitled to a period of consideration before you decide whether to sign the consent declaration.

Purpose of the research

During admission on the intensive care unit, we routinely treat our patients with a low dose anticoagulant (enoxaparin) to prevent blood clots in the deep veins. Intensive care patients who suffer from acute kidney failure, are at the greatest risk of developing blood clots in the deep veins. Studies suggest that the standard dose (enoxaparin 40 mg) is too low for patients admitted to the intensive care unit.

Type of research intervention

We would like to treat you with a standard dose (enoxaparin 40 mg) or a moderately increased dose (enoxaparin 1mg/kg) anticoagulant. The dose assigned to each participant is selected randomly, that is to say, it is left to chance if you are treated with the standard dose or a moderately increased dose. This involves choosing an opaque envelope which is then given to the participant's nurse who will then administer the indicated dose. The trial will start once dialysis is commenced, and will continue until discharge from the intensive care department.

Participant selection

We are inviting all adults on the intensive care unit who develop acute kidney failure and are on dialysis to participate. The following conditions render patients ineligible for participation: allergy to enoxaparin, pregnancy, presence of chronic renal failure, and presence of low platelets or uncontrolled bleeding.

Voluntary participation

It is voluntary to participate in the trial. You can, at any time and without cause, withdraw your consent. If you do not want to participate, this will not affect your right to any current or future treatment or any other rights during your stay on the intensive care unit. If you choose not to participate in this research project, you will receive the treatment that is routinely offered in this hospital for prevention of blood clots. You may change your mind later and stop participating even if you agreed earlier.

Information on enoxaparin

A rare side effect of enoxaparin is oozing from injection sites and a decrease in the number of platelets. If either of these signs occurs, you will be evaluated by a doctor to decide whether you should discontinue the trial.

Procedures and Protocol

We will take weekly blood samples from the vascular access lines, and urine samples from a urinary catheter, both of which would have been placed during your stay on the intensive care unit. The samples will be preserved in a research biobank, analysed within 3 years, and discarded after the project is completed. The samples obtained during this trial will only be used for this research, and will be destroyed after 3 years, when the research is completed.

You will be assessed on a daily basis for (i) bleeding, and (ii) blood clots in the deep veins. On suspicion of blood clots in the deep veins, you will undergo an ultrasound of both legs. Ultrasound of the heart and a CT scan of the lungs will be performed on suspicion of a blood clot in the lungs.

We suspect, but do not yet know that a moderately increased dose of anticoagulant as opposed to the standard dose will better prevent blood clots. To confirm this suspicion, we need to compare the two doses. To do this, we will put people taking part in this research into two groups. The groups are selected by chance, as if by tossing a coin. Participants in one group will be given enoxaparin 40 mg, while participants in the other group will be given enoxaparin 1mg/kg. We will then compare which of the two has the best results.

Duration

266 patients will be included over 3 years.

Side Effects

We will follow you closely and keep track of any unwanted effects or problems. Side effects of enoxaparin include oozing from injection sites and a decrease in the number of platelets. If either of these signs occurs, you will be evaluated by a doctor to decide whether you should discontinue. If we detect other side effects, you will be informed immediately, and you will have to decide whether you want to continue in the study. The treatment will be adjusted based on the department's standard principles and in accordance with the project's protocol in the case of: dialysis free periods, bleeding, blood clots, side effects, or the termination of the project.

Risks

By participating in this research it is possible that you will be at increased risk of oozing. When this occurs, it is the responsibility of the attending physician to decide whether it is related to enoxaparin.This risk is assessed as minimal due to the low dose used, and because the activity in the blood will be monitored. Fresh frozen plasma will be administered with signs of oozing from injection site. Serious and unexpected adverse reactions will be reported to the Danish Health and Medicines Authority.

Benefits

The study will benefit you, because in contrast to the department's routine, participants will be screened for blood clots in the deep veins, and ultrasound of both legs and heart will be performed. If we can reduce the incidence of blood clots in the deep veins through the optimization of enoxaparin treatment, it will reduce the number of deaths caused by these clots and hopefully influence therapy of critically ill dialysis patients worldwide.

Compensation

With the trial, there is the possibility to appeal and receive compensation according to the Act on the Right to Complain and Receive Compensation within the Health Service.

Confidentiality

Records of each participant's identity and personal information will not be made public. Employees from Good Clinical Practice, Danish Health and Medicines Authority and the corresponding foreign authority will have access to your medical records and information collected in connection with the study for quality control (monitoring, audit and inspection) of the project. Danish Health and Medicines Authority will have access to scientific data and patient records for up to 5 years after the trial.

Sharing the Results

Six months after the end of the trial, you can get information on the outcome by contacting Professor Palle Toft. The trial is registered in the EU Clinical Trials Register. The research findings will be shared through publications and national/international conferences. Strict confidentiality will be observed.

Alternatives to Participating

If you do not wish to take part in the trial, you will be provided with the established standard treatment available for preventing blood clots on the intensive care unit. Enoxaparin administered at a standard dose of 40 mg, is the department's standard treatment for the prevention of blood clots in the deep veins.

Information about our sponsors:

The trial is being funded by the Danish Society of Anaesthesiology & Intensive Medicine's research initiative, Odense University Hospital's research grant, and the Lippmann Fund.

Who to contact:

The study was approved by the Danish national scientific ethical committee and the Danish Health and Medicines Authority.

We hope that with this information you have gained sufficient insight into what it means to participate in this trial, and that you feel equipped to make decisions about your possible participation. We ask that you also read the accompanying literature "The subject's rights in a biomedical research project". To learn more about the study, or if you have any questions, please feel free to contact us:

Principal Investigator
Sian Robinson, M.B., B.S.
Odense University Hospital
Dept. of Anaesthesiology and Intensive Care
Sdr. Boulevard 29. Odense C
DK 5000. Denmark
Telephone: +45 6541 5519
Email: sian.robinson@rsyd.dk

Sponsor-Investigator
Professor Palle Toft
Odense University Hospital
Dept. of Anaesthesiology and Intensive Care
Sdr. Boulevard 29. Odense C
DK 5000. Denmark
Telephone: +45 6541 3947
Email: palle.toft@rsyd.dk
