# Supplementary material for: A feasible strategy for preventing blood clots in critically ill patients with acute kidney injury (FBI): study protocol for a randomized controlled trial
Source: Trials. 2014 Jun 13;15:226. doi: 10.1186/1745-6215-15-226 (PMC4061539; doi:10.1186/1745-6215-15-226)
Supplement: Additional file 3 — Informed consent for participation in the biomedical research project: a feasible strategy for preventing blood clots in critically ill patients with acute kidney injury (FBI). [file 1745-6215-15-226-S3.pdf]

OUH
Odense
Universitetshospital

Region Syddanmark

Informed Consent for participation in the biomedical research project: A feasible strategy for preventing blood clots in critically ill patients with acute kidney injury (F.B.I.)

I have read the foregoing information, or it has been read to me. I have had the opportunity to ask questions about it and any questions that I have asked have been answered to my satisfaction. I am aware that my participation in the trial is voluntary, and that I can, at any time and without cause, withdraw my consent. I am also aware that refusal to participate will not affect any current or future treatment. I will be offered the treatment that is routinely offered in this hospital for prevention of blood clots.

I consent voluntarily to participate as a participant in this research, and consent to my biological samples obtained during this trial being preserved in a biobank.

Print Name of Participant__________________

Signature of Participant ___________________

Date ___________________________

Day/month/year

Would you like to receive information on the outcome and the eventual consequence for you?

Yes ☐ **No** ☐

Statement by the researcher taking consent:
I have accurately read out the information sheet to the potential participant, and to the best of my ability made sure that the participant understands what the project entails. I confirm that the participant was given the opportunity to ask questions about the study, and all the questions asked by the participant have been answered correctly and to the best of my ability. I confirm that the individual has received sufficient information to allow insight into what it means to participate in the trial, and to feel equipped to make decisions about participation. A copy of this ICF has been provided to the participant, along with written material about the project.

Signature of Researcher taking the consent__________________________

Date ___________________________

Day/month/year

Protocol: A feasible strategy for preventing blood clots in critically ill patients with acute kidney injury (F.B.I.)
Version 5. EudraCT-nummer: 2012-004368-23 Danish National Scientific Ethical Committee: 1210528
Danish Health and Medicines Authority: 2012100176
