# Supplementary material for: A feasible strategy for preventing blood clots in critically ill patients with acute kidney injury (FBI): study protocol for a randomized controlled trial
Source: Trials. 2014 Jun 13;15:226. doi: 10.1186/1745-6215-15-226 (PMC4061539; doi:10.1186/1745-6215-15-226)
Supplement: Additional file 4 — Report form for Serious Adverse Event and Suspected Unexpected Serious Adverse Event. [file 1745-6215-15-226-S4.pdf]

Report form for SAE/SUSAR:

**PART 1**: SAE Serious Adverse Event Report (from Investigator to Sponsor)<br>**PART 2**: SUSAR Suspected Unexpected Serious Adverse Event Report (Sponsors assessment)

Protocol title: A feasible strategy for preventing blood clots in critically ill patients with acute kidney injury (F.B.I.)- prospective randomised, double-blinded multicentre study

EudraCT-nummer: 2012-004368-23; SST 2012100176; DNVK1210528

PART 1 (To be filled in by Investigator)

Report date: ______________________________
Day/month/year

Report type:

Initial □	Follow up □

Subject Information:

Patient initials	Country	Date of birth: ______________________________ Day/month/year	Sex<br>M □ F □	Height:<br><br>Weight:
Patient no.	Site no.			

Serious Adverse Event:

SAE: (diagnose)	
SAE Onset date:<br>______________________________<br>Day/month/year	SAE End date:<br>______________________________<br>Day/month/year
Patient discontinued from study due to SAE<br>Yes □ → date: ______________________________ No □<br>Day/month/year	

SAE Criteria **Evaluation**

**Serious Criteria**	**Outcome**	**Severity**	**Action Taken**
Patient died □<br><br>Life threatening □<br><br>Involved persistence of significant disability or incapacity □<br><br>Involved prolonged inpatient hospita-lisation □<br><br>Important Medical Event □<br><br>Other □	Ongoing □<br><br>Resolved □<br><br>Resolved with Sequelae □<br><br>Fatal □<br><br>Unknown □	Grade 1 □<br><br>Grade 2 □<br><br>Grade 3 □<br><br>Grade 4 □	No change □<br><br>Drug dose changed □<br><br>Drug temporarily discontinued □<br><br>Drug permanently discontinued □

Death:

Date of death<br><br>____________________<br>Day/month/year	Cause of death
Death certificate<br><br>Yes (attach copy) □ No □	Autopsy report<br><br>Yes (attach copy) □ No □

Relationship to study drug:

Unrelated to study drug □<br>(None, unlikely)	Related to the study drug □<br>(Possible, probable, definite)

Suspect Drug(s) information:

Suspect Drug(s) name:<br><br>Batch no:	Did reaction abate after stopping drug?<br>Yes ◻ No ◻ NA ◻
Daily dose(s) (specify units)	Route(s) of administration
Indications(s) for use	Did reaction reappear after reintroduction?<br>Yes ◻ No ◻ NA ◻
Therapy starting date<br><br>__________________________<br>Day/month/year	Therapy stopping date<br><br>__________________________<br>Day/month/year

Event Description:

Concomitant Medication(s) relevant to the event (exclude those used to treat event):

Concomitant drug(s) and dates (Day/month/year) of administration.

**Reporter information**	**Investigator information**
Name:	Name:
Address:	Address:
Phone:	Phone:
Profession:	Profession:
Signature & Date	Signature & Date

Fill in this form and fax it within 24 hours to:

Sponsor: Professor Palle Toft

Fax number: +45 66 11 34 15
Telephone number: +45 65 413947

Sponsors date and signature for receiving this report:

Date Signature

PART 2: (To be filled in by sponsor)

Causality Assessment by Sponsor:

Result of causality evaluation:

□ Not related to study drug (Unlikely/doubtful) → (If judged related by investigator go to box below)
□ Related to study drug (Possible/Probable/Definite) → (Go to box below)

Expectedness Assessment by Sponsor (only relevant if SAE is related to study drug):

Result of expectedness evaluation:

□ Expected (due to relevant reference document)
□ Unexpected→ (Go to box below)

Summary:

Category of event:

□ SUSAR (SAE is both related and unexpected)
□ SAR (SAE is related but not unexpected)
□ SAE (SAE is not related)

Notify relevant authorities according to protocol

Sponsors comments (including information regarding unblinding):

Sponsors date and signature:

Date Signature
