# Supplementary material for: A feasible strategy for preventing blood clots in critically ill patients with acute kidney injury (FBI): study protocol for a randomized controlled trial
Source: Trials. 2014 Jun 13;15:226. doi: 10.1186/1745-6215-15-226 (PMC4061539; doi:10.1186/1745-6215-15-226)
Supplement: Additional file 5 — Definitions. [file 1745-6215-15-226-S5.pdf]

Protocol: A feasible strategy for preventing blood clots in critically ill patients with acute kidney injury (F.B.I.)

Definitions:

Renal recovery: as defined in this protocol refers to the complete independence from renal replacement therapy after the first dialysis-free interval.

Deep vein thrombosis (DVT): occurs when a venous segment cannot be partially or totally compressed.

Pulmonary embolism (PE): occurs when there is an intra-luminal filling defect on spiral pulmonary CT angiography, or is detected at autopsy.

Venous thromboembolism (VTE): include PE, and DVT of the proximal lower limb.

Catheter-related thrombus: is the presence of intravascular catheter in the same / adjacent venous segment for up to 3 days prior to diagnosis.

Major bleeding:

- bleeding from a critical site (e.g. intracranial, pericardial)
- bleeding leading to haemodynamic instability
- bleeding requiring treatment (e.g. surgery, transfusion of > 2 units of packed red blood cells)
- fatal bleeding - the patient died because of bleeding and not with bleeding

Minor bleeding : bleeding that does not meet the criteria for major or fatal bleeding [11].

Bioaccumulation: is defined by detection of at least 1 trough level of anti-factor Xa > 0.40 IU/ml [6].
