# Supplementary material for: A feasible strategy for preventing blood clots in critically ill patients with acute kidney injury (FBI): study protocol for a randomized controlled trial
Source: Trials. 2014 Jun 13;15:226. doi: 10.1186/1745-6215-15-226 (PMC4061539; doi:10.1186/1745-6215-15-226)
Supplement: Additional file 6 — Conduct of continuous renal replacement therapy. [file 1745-6215-15-226-S6.pdf]

**Protocol:** A feasible strategy for preventing blood clots in critically ill patients with acute kidney injury (F.B.I.)

Conduct of continuous renal replacement therapy:

Participants will be treated with continuous renal replacement therapy (CRRT) using either:

1) multiFiltrate haemodialysis system with ultraflux AV 1000S polysulphone filter (surface area 1.8 m2) and replacement fluid multiBic (all from Fresenius Medical Care, Bad Homburg, Germany) – at Odense University Hospital

**or**

2) aquarius™ haemodialysis system with aquamax HF12 polyethersulfone filter (surface area 1.2 m2 ) and replacement fluid Accusol 35 (all from Baxter A/S, Allerød , Denmark) – at Svendborg Hospital and SVS Esbjerg Hospital.

Patients will commence dialysis in continuous veno-venous haemofiltration (CVVH) modus with a blood flow of 300 ml/minute at all institutions. The prime fluid is isotonic saline with 5000 IU/L heparin. Replacement fluid of 35 ml/ kg/hour will be delivered pre- and postfilter at an initial ratio of 1:1. Fluid removal rates are left to the discretion of the attending physician in order to achieve optimal haemodynamic balance. The attending physician will also dictate the concentration of potassium chloride to be used in the replacement fluid.

No anticoagulation will be used initially. With an alarm for high pressure, blood flow and pre- and postdilution will be adjusted (the latter to 70% pre- and 30% postdilution). If a filter time > 18-24 hours is not possible, infusion unfractionated heparin (UFH) 5-10 IU/kg/hour will be commenced at all institutions. With continuing clotting, prostacyclin (at SVS Esbjerg and Svenborg hospitals) or regional citrate (at Odense University Hospital) will be used, unless there are contraindications.

A change to citrate at Odense University Hospital, will require a change in CRRT modus to continuous veno-venous haemodialysis (CVVHD). Flow rates of blood and dialysate fluid will be adopted to the equivalent of ~4 mmol citrate per L and adapted to the patient's weight. Calcium losses caused by the extracorporeal clearance of citrate–calcium complexes will be substituted by an intravenous infusion of a 500 mmol/L calcium chloride solution. Postfilter ionised calcium levels will be maintained between 0.25 and 0.34 mmol/L.

The use of diuretics is prohibited under CRRT, but furosemide at a maximum dose of 5 mg/hour is allowed in CRRT free intervals.

CRRT will be discontinued if the urine volume exceeds 200ml/day (without the aid of diuretics). CRRT will be resumed if the patient has: a) oliguria (diuresis <200 ml for 24 hours), b) anuria (diuresis < 50 ml for 12 hours), c) creatinine clearance < 12 ml/min/1.73 $m^2$ d) hyperkalemia ( serum-potassium > 6.0 mmol/l) unresponsive to conventional therapy) e) severe metabolic acidosis ( pH < 7.15), or f) respiratory distress due to overhydration (judged clinically from an increase in PEEP of 5 cm H2O and/or radiological evidence of lung oedema).
